# Risk factors for multisystem inflammatory syndrome in children – A population-based cohort study of over 2 million children

**DOI:** 10.1016/j.lanepe.2022.100443

**Published:** 2022-06-22

**Authors:** Samuel Rhedin, Cecilia Lundholm, AnnaCarin Horne, Awad I. Smew, Emma Caffrey Osvald, Araz Haddadi, Tobias Alfvén, Robin Kahn, Petra Król, the Swedish Pediatric MIS-C Consortium, Bronwyn Haasdyk Brew, Catarina Almqvist

**Affiliations:** a Department of Medical Epidemiology and Biostatistics, Karolinska Institutet, Stockholm, Sweden; b Sachs’ Children and Youth Hospital, Stockholm, Sweden; c Department of Women's and Children's Health, Karolinska Institutet, Stockholm, Sweden. & Department of Paediatric Rheumatology, Astrid Lindgren Children's Hospital, Karolinska University Hospital, Stockholm, Sweden; d Pediatric Allergy and Pulmonology Unit at Astrid Lindgren Children's Hospital, Karolinska University Hospital, Stockholm, Sweden; e Department of Global Public Health, Karolinska Institutet, Stockholm, Sweden; f Department of Paediatrics, Clinical Sciences Lund, Lund University, Lund, Sweden; g Wallenberg Centre for Molecular Medicine, Lund University, Lund, Sweden; h National Perinatal Epidemiology and Statistics Unit, Centre for Big Data Research in Health, University of NSW, Sydney, Australia

**Keywords:** MIS-C, COVID-19, Risk Factors, Cohort study, Pediatric infectious diseases, Epidemiology

## Abstract

**Background:**

Although severe acute COVID-19 is rare in children, SARS-CoV-2 infection can trigger the novel post-infectious condition multisystem inflammatory syndrome in children (MIS-C). Increased knowledge on risk factors for MIS-C could improve our understanding of the pathogenesis of the condition and better guide targeted public health interventions. The aim of the study was to assess risk factors for MIS-C with the aim to identify vulnerable children.

**Methods:**

A register-based cohort study including all children and adolescents <19 years born in Sweden between March 1, 2001- December 31, 2020 was performed. Data on sociodemographic risk factors and comorbidities (sex, age, parental region of birth, parental education, asthma, autoimmune disease, chromosomal anomalies, chronic heart disease, chronic lung disease, obesity, life-limiting condition) were retrieved from national health and population registers. The outcome was MIS-C diagnosis according to the Swedish Pediatric Rheumatology Quality Register during March 1, 2020 – December 8, 2021.

Hazard ratios (HRs) and 95% confidence intervals (CIs) were calculated using Cox regression analysis. Incidence rates per 100 000 person-years were calculated assuming a Poisson distribution.

**Findings:**

Among 2 117 443 children included in the study, 253 children developed MIS-C, corresponding to an incidence rate of 6·8 (95% CI: 6·0-7·6) per 100 000 person-years. Male sex (HR 1·65, 95% CI: 1·28-2·14), age 5-11 years (adjusted HR 1·44, 95% CI: 1·06-1·95 using children 0-4 years as reference), foreign-born parents (HR 2·53, 95% CI: 1·93-3·34), asthma (aHR 1·49, 95% CI: 1·00-2·20), obesity (aHR 2·15, 95% CI: 1·09-4·25) and life-limiting conditions (aHR 3·10, 95% CI: 1·80-5·33) were associated with MIS-C. Children 16-18 years had a reduced risk for MIS-C (aHR 0·45, 95% CI: 0·24-0·85).

**Interpretation:**

We report increased risks for MIS-C in children with male sex, age 5-11 years, foreign-born parents, asthma, obesity, and life-limiting condition. Knowing these risk populations might facilitate identification of children with MIS-C and potentially guide targeted public health interventions. Nevertheless, the absolute risks for MIS-C were very low.

**Funding:**

Financial support was provided from the Swedish Research Council (grant no 2018-02640), the Swedish Heart-Lung Foundation (grant no 20210416), the Asthma and Allergy Association, Ake Wiberg foundation, the Samariten Foundation, the Society of Child Care, and Region Stockholm.

**Research in context**

**Evidence before this study**

While severe acute COVID-19 is rare in children, SARS-CoV-2 infection can trigger the novel post-infectious inflammatory condition multisystem inflammatory syndrome in children (MIS-C). Little is known on risk factors for developing MIS-C. Previous case series and surveillance studies of MIS-C have reported an overrepresentation of males, younger school-aged children, children with black non-Hispanic ethnicity and pediatric chronic complex conditions, but there is limited data from cohort studies on risk factors for developing MIS-C, in particular on the role of comorbidities.

**Added value of this study**

In this population-based register study of >2 million Swedish children 253 children developed MIS-C, corresponding to an incidence rate of 6·8 (95% CI: 6·0-7·6) per 100 000 person-years. We report that male sex, age, foreign-born parents, asthma, obesity, and life-limiting conditions are associated with MIS-C.

**Implications of all the available evidence**

Knowing these risk populations might facilitate identification of children with MIS-C and potentially guide targeted public health interventions. Nevertheless, the absolute risks for MIS-C were very low.

Alt-text: Unlabelled box

## Background

While COVID-19 is associated with substantial mortality in the elderly, severe acute disease is rare in children.^1^ In spring 2020, however, a novel post-infectious inflammatory condition associated with COVID-19 was described in children.^2^ This condition, later termed multisystem inflammatory syndrome in children (MIS-C), is characterized by prolonged fever, gastrointestinal symptoms, and rash, thus to some extent resembling Kawasaki disease.^3^^,^^4^ MIS-C, also termed PIMS-TS (Paediatric multisystem inflammatory syndrome temporally associated with COVID-19), has been defined by the World Health Organization.^5^ In brief, MIS-C should be considered in individual under 19 years with persistent fever more than 3 days, signs and symptoms consistent with multisystem inflammation involving ≥2 organ systems, a history of SARS-CoV-2 infection and other disorders excluded. MIS-C is a rare but severe condition that requires immunomodulatory treatment and can result in acute heart failure, coronary artery dilatation or arrhythmias.^6^ Case series and surveillance studies of MIS-C patients in the United States have reported an overrepresentation of males, younger school-aged children, children with black non-Hispanic ethnicity and pediatric complex chronic conditions.^6^, ^7^, ^8^, ^9^ On the other hand, studies in adults have identified asthma, autoimmune disease, cardiovascular disease, obesity as risk factors for severe COVID-19 disease.^10^^,^^11^

Large studies have reported that vaccination against SARS-CoV-2 reduces the risk of developing MIS-C.^12^ In Sweden, COVID-19 vaccination in adolescents started in summer 2021 and is currently recommended for all children and adolescents ≥12 years. In children 5-11 years only at-risk populations for presumed severe viral respiratory tract infections or severe COVID-19 are recommended COVID-19 vaccination (severe asthma previously requiring ICU-treatment, severe heart/lung/neurological/rheumatological disease, severe primary and secondary immune deficiencies/certain previous organ/stem cell transplantations, severe obesity or trisomy 21 with history of susceptibility to severe infections).^13^ There is a need for population-based studies on risk factors for MIS-C to be able to identify vulnerable children that might benefit from targeted public health interventions such as COVID-19 vaccination campaigns. An increased knowledge of risk factors for MIS-C could also improve our understanding of the pathogenesis of MIS-C.

The aim of this study was to assess risk factors for MIS-C in a large register-based study of the entire Swedish child and adolescent population.

## Methods

### Study design and population

A register-based cohort study was performed by retrieving data from the Total Population Register (TPR) on all Swedish children and adolescents (henceforth referred to as children) born March 1, 2001- December 31, 2020, to make sure they were <19 years when community spread of COVID-19 started in Sweden. Exclusion criteria were death or emigration before March 1, 2020, being born abroad, or missing a link to the mother's identifier. Each Swedish citizen holds a unique identifier that can be used to link different national health and quality registers with nearly 100% accuracy.^14^ In this study we linked the total population register to the following registers: I) the National Patient Register (NPR) that contains International Classification of Disease 10^th^ Revision (ICD-10) codes for all hospital visits and approximately 80% of visits to specialist outpatient units; II) the Swedish Prescribed Drug Register (SPDR) that contains data on all dispensed medications since July 2005; III) the Medical Birth Register (MBR) that contains data on perinatal parameters including maternal smoking during pregnancy; IV) the Longitudinal Integrated Database for Labor Market Studies (LISA) containing data on education and region of birth; V) the Multi-Generation Register, containing data on the parent's identities; VI) the Swedish Pediatric Rheumatology Quality Register (pedSRQ) containing data on children with MIS-C; and VII) the Child Obesity Quality Register BORIS that contains records of interventions and treatments for obesity and overweight in children since 2005. We retrieved data from the MBR until December 31, 2019, for pedSRQ until December 8, 2021, and until December 31, 2020, for the other registers (Figure S1).

### Study variables

The outcome was defined as a record of MIS-C in the pedSRQ. Since the first MIS-C case was reported in Sweden on May 13, 2020, the Swedish Pediatric Rheumatology Association initiated biweekly meetings, with representatives from most parts of Sweden, discussing all new MIS-C cases and implemented mandatory reporting of MIS-C, i.e., before the ICD-10 code for MIS-C was available.

Sociodemographic characteristics and comorbidities were assessed as potential risk factors. Age groups were categorized as 0-4 years, 5-11 years, 12-15 years, and 16-18 years, to align with previous COVID-19 vaccine safety studies.^15^^,^^16^ Data on sex, parental region of birth (classified as both parents born in Sweden, one parent born in Sweden or both parents born abroad) and educational level (classified as highest parental educational level as of December 31, 2019: primary school (0-9 years), secondary school (12 years) or tertiary education (>12 years)) were retrieved from LISA and TPR.

Asthma was defined according to a previously validated algorithm based on both records of asthma diagnosis in the NPR and prescription of asthma medications in the SPDR.^17^ Obesity was defined as a record of ICD-10 codes E60-E65 in the NPR or a record of obesity grade I-III in the BORIS register. The other comorbidities were solely defined according to records of specific ICD-10 codes in the NPR (see Table S1 for details on ICD-10 codes used).^18^ For all the comorbidities, only diagnoses recorded before March 1, 2020, were considered to ensure that the comorbidity was not identified during the diagnostic work-up for the MIS-C diagnosis. Life-limiting condition was defined as conditions associated with a significantly shorter life expectancy and for which there is no available curative treatment as previously established by Fraser *et al*.^19^ (Table S1).

Data on maternal smoking during pregnancy and number of siblings were retrieved from the MBR and the Multi-Generation Register and included as potential confounders in some of the adjusted models (Figure S2).

### Statistical analyses

Hazard ratios (HRs) and 95% confidence intervals (CIs) for MIS-C were estimated for each risk factor using multivariate Cox regression analysis adjusting for potential confounders. Study subjects were censored in the analyses when they turned 19 years, emigrated/died or were diagnosed with MIS-C. When assessing the association between age and MIS-C, the proportional hazards assumption was tested based on Schoenfeld residuals, which indicated non-proportionality (p<0.01). Hence, we allowed for time-varying effects, assuming piecewise constant hazards for the period March 1 - October 12^,^ 2020 (i.e. the first MIS-C wave) and October 13, 2020 – December 8, 2021, respectively (the second and third MIS-C wave) based on graphical examination of the log hazard curves and the incidence of MIS-C.

Age, sex, parental region of birth, and parental education were assessed in the full cohort, whereas comorbidities (except for obesity) were assessed in the comorbidity cohort only including children born prior to the SARS-CoV-2 outbreak (i.e. children born before March 1, 2020). Finally, obesity was assessed in the obesity cohort further excluding children who were <5 years at March 1, 2020, were excluded since obesity is rarely diagnosed before 4 years of age in Sweden.^20^

Potential confounders were selected separately for each studied risk factor by creating directed acyclic graphs based on the literature taking into account the possibility of multicollinearity (Figure S2).^21^ For sex and parental origin, no plausible confounders were identified, and hence these risk factors were only assessed in unadjusted analyses. For analyses with adjustment for age, we used stratified Cox regression with stratification by age to handle the age-related non-proportional hazards. Incidence rates per 100 000 person-years and 95% CIs were estimated for each of the studied risk factors, assuming a Poisson distribution.

A power calculation was performed assuming an estimated n=270 MIS-C cases, a study population of n=2 000 000 children at an alpha level of 0·05 and 80% power. This resulted in a need for approximately 49 000 exposed children for each specific risk factor to be able to detect a risk ratio of 2·5 and approximately 33 000 exposed children to be able to detect a risk ratio of 3. As life-limiting conditions represent a heterogeneous group of medical conditions that partly overlapped with some of the other studied comorbidities, these children were excluded in a sensitivity analysis. To account for multiple testing, Bonferroni corrected p-value threshold were calculated assuming 11 tests and an overall significance level of 5% (p-value threshold=0·0045). Data analysis was performed in Stata version 16·1 (StataCorp, College Station, TX, USA). The study was approved by the Swedish Ethical Review Authority (DNR 2018/1697-31/1).

### Role of the funding source

The funders had no role in the design and conduct of the study.

## Results

**Figure 1:**
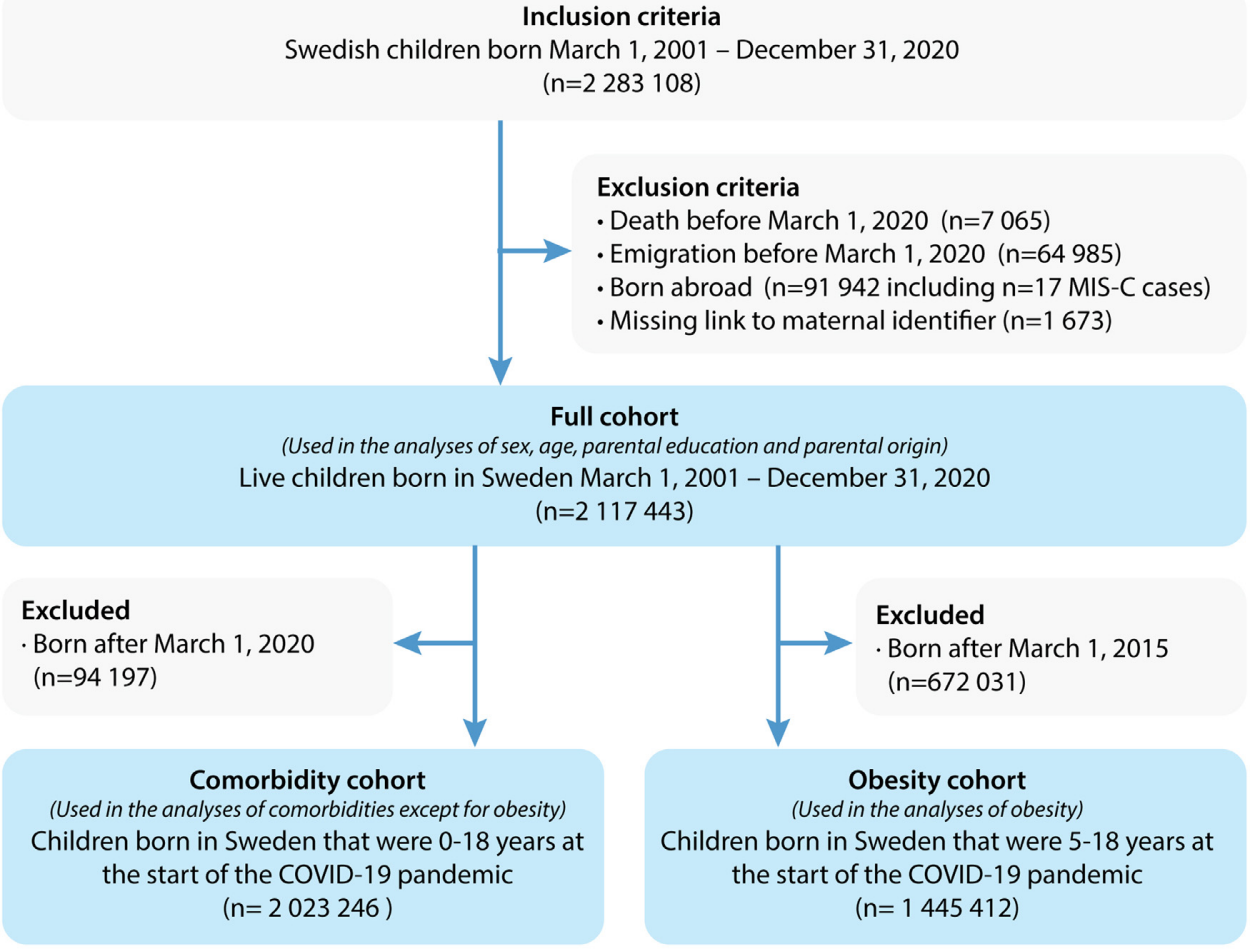
Flowchart of study cohorts.

A total of 2 283 108 children were identified during the study period. Children who died (n=7 065), emigrated before March 1^st^ 2020 (n=64 985), were born abroad (n=91 942) or had missing link to the mother's identifier (n=1 673) were excluded. Hence, n=2 117 443 children that were <19 years on March 1, 2020, were included in the study (Figure 1). There were 556 872 (26·3%) children 0-4 years, 882 787 (41·7%) children 5-11 years, 401 044 (28·9%) children 12-15 years and 276 740 (13·1%) children 16-18 years (Table 1). Asthma was the most common disease of all the studied comorbidities (n=194 796, 9·2%), followed by chronic heart disease (n=49 471, 2·3%). Chronic lung disease (n=11 044, 0·5%) and chromosomal anomalies (n=5 361, 0·3%) were the least common comorbidities.

**Table 1:** Characteristics of study population.

Characteristic	0-4 years (n = 556 872)	5-11 years (n = 882 787)	12-15 years (n = 401 044)	16-18 years (n = 276 740)	All (n = 2 117 443)
Male sex	286 370 (51·4)	453 893 (51·4)	206 299 (51·4)	142 333 (51·4)	1 088 895 (51·4)
Maternal smoking during pregnancy	17 491 (4·4)	49 634 (6·1)	28 482 (7·9)	26 632 (10·3)	122 239 (6·6)
Siblings, mean (SD)	1·1 (1·1)	1·5 (1·0)	1·4 (1·1)	1·0 (1·1)	1·3 (1·1)
Highest parental education					
Primary school (9 years)	31 739 (5·8)	36 252 (4·1)	11 562 (2·9)	7 845 (2·8)	87 398 (4·2)
Secondary school (12 years)	177 524 (32·4)	281 647 (32·1)	136 820 (34·2)	104473 (37·8)	700 464 (33·3)
Tertiary education (>12 years)	338 810 (61·8)	559 769 (63·8)	251 369 (62·9)	163 767 (59·3)	1 313 715 (62·5)
Parental region of birth					
Both parents born in Sweden	347 345 (62·4)	606 312 (68·7)	298 997 (74·6)	212 806 (76·9)	1 465 460 (69·2)
One parent born in Sweden	82 615 (14·8)	123 740 (14·0)	50 495 (12·6)	32 572 (11·8)	289 422 (13·7)
Both parents born abroad	126 912 (22·8)	152 735 (17·3)	51 552 (12·9)	31 362 (11·3)	362 561 (17·1)
Comorbidities					
Asthma	6 033 (1·1)	98 436 (11·2)	53 172 (13·3)	37 155 (13·4)	194 796 (9·2)
Autoimmune disease	409 (0·1)	7 120 (0·8)	8 830 (2·2)	10 444 (3·8)	26 803 (1·3)
Chromosomal anomalies	751 (0·1)	2 409 (0·3)	1 295 (0·3)	906 (0·3)	5 361 (0·3)
Chronic lung disease	2 815 (0·5)	5 245 (0·6)	1 765 (0·4)	1 219 (0·4)	11 044 (0·5)
Chronic heart disease	11 168 (2·0)	22 281 (2·5)	9 717 (2·4)	6 305 (2·3)	49 471 (2·3)
Life-limiting condition	5 876 (1·1)	19 014 (2·2)	11 025 (2·8)	8 510 (3·1)	44 425 (2·1)
Obesity	611 (0·1)	15 643 (1·8)	13 040 (3·3)	9 347 (3·4)	38 641 (1·8)

Number presented as n (column %) if not otherwise specified.

There were 253 records of MIS-C during the study period corresponding to an incidence rate of 6·8 (95% CI: 6·0-7·6) per 100 000 person-years.

### Sociodemographic factors and risk for MIS-C

**Figure 2:**
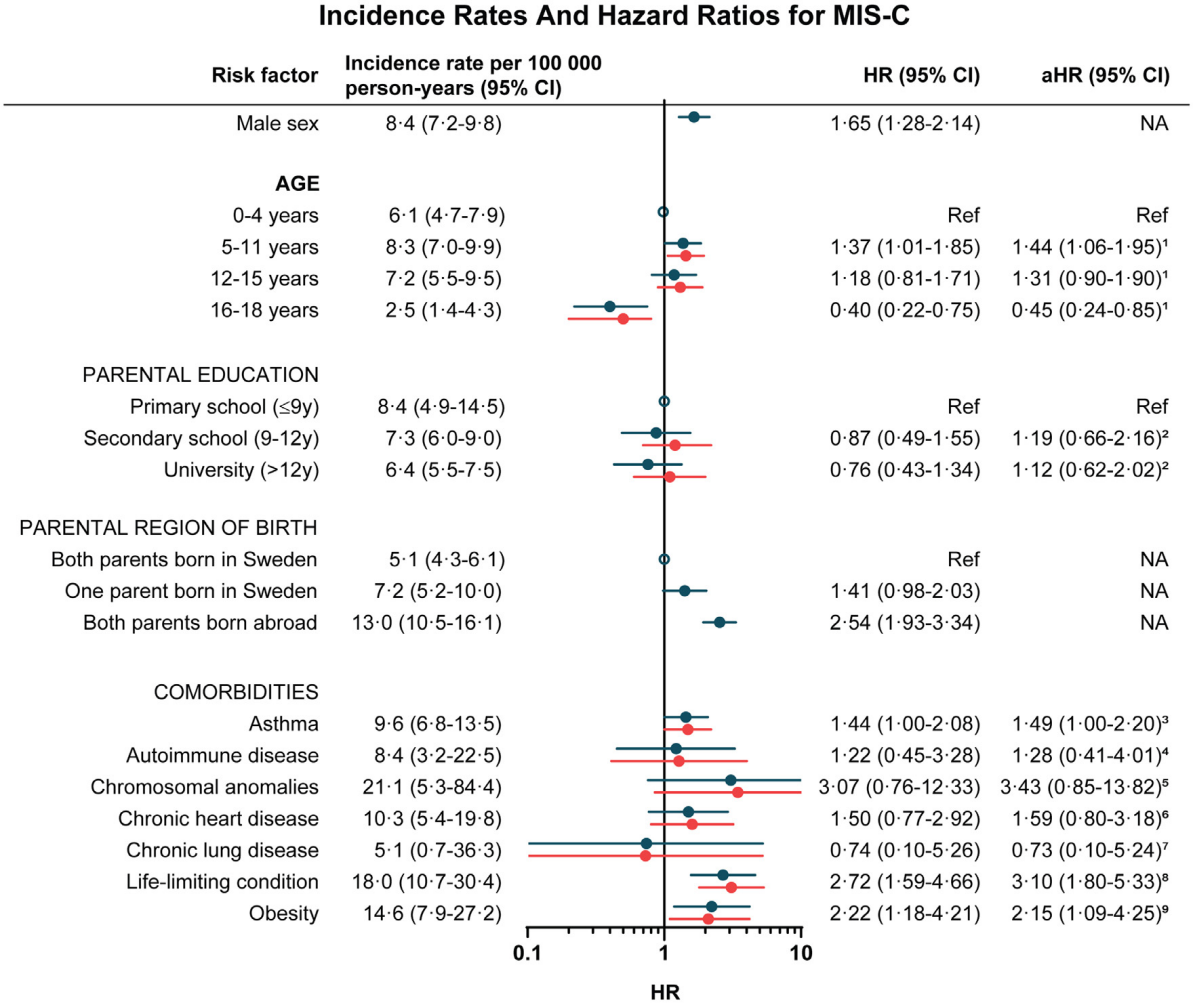
Risk factors for MIS-C in children. Crude (blue) and adjusted (red) hazard ratios for MIS-C. ^1^Adjusted for parental education, parental region of birth. ^2^Adjusted for parental region of birth, siblings. ^3^Adjusted for age, sex, parental education, parental region of birth siblings, maternal smoking during pregnancy, chronic lung disease. ^4^Adjusted for age, sex, parental education, parental region of birth, siblings, maternal smoking during pregnancy. ^5^Adjusted for age, sex, parental education, parental region of birth, siblings, maternal smoking during pregnancy. ^6^Adjusted for age, sex, parental education, parental region of birth, maternal smoking during pregnancy, chromosomal anomalies. ^7^Adjusted for age, sex, parental education, parental region of birth, maternal smoking during pregnancy, asthma. ^8^Adjusted for age, sex, parental education, parental region of birth, maternal smoking during pregnancy. ^9^Adjusted for age, sex, parental education, parental region of birth, maternal smoking during pregnancy. Abbreviations: CI, confidence interval; HR, hazard ratio; MIS-C, multisystem inflammatory syndrome in children; NA, not applicable.

Age was significantly associated with MIS-C with a higher risk in children 5-11 years (aHR 1·44, 95% CI: 1·06-1·95) and a lower risk in children 16-18 years (aHR 0·45, 95% CI: 0·24-0·85) as compared to children 0-4 years (Figure 2). Further, male sex (HR 1·65, 95% CI: 1·28-2·14) and foreign-born parents (HR 2·54, 95% CI: 1·93-3·34) were associated with MIS-C. Of all sociodemographic factors, the highest absolute risk was seen in children with foreign-born parents with an incidence rate of 13.0 (95% CI: 10.5-16.1) per 100 000 person-years. Parental education was not associated with MIS-c (Figure 2).

There was a tendency of interaction between SARS-CoV-2 waves and age with regard to risk for MIS-C, with a major difference in children 12-15 years (HR 2·20, 95% CI 1·07-4·53 during the first COVID-19 wave and HR 0·92, 95% CI: 0·59-1·44 during the second and third COVID-19 wave, test for non-proportional hazards p=0·052) (Table S2).

### Comorbidities and risk for MIS-C

Having asthma (aHR 1.49, 95% CI: 1.00-2.20), obesity (aHR 2.15, 95% CI: 1.09-4.25) or a life-limiting condition (aHR 3.10, 95% CI: 1.80-5.33) were each significantly associated with MIS-C (Figure 2). The associations observed for asthma and obesity, however, did not remain statistically significant when adjusting for multiple testing using the Bonferroni method (Table S3). There were minor changes in the estimates in the sensitivity analysis where children with life-limiting conditions were excluded (Table S4). Having foreign-born parents (aHR 2·62, 95% CI: 1·97-3·47) remained the risk factor most strongly associated with MIS-C.

## Discussion

In this large population-based cohort study we assessed risk factors for MIS-C in >2 million Swedish children and adolescents. The overall risk for MIS-C was very low but we report increased risks for MIS-C in children of male sex, with foreign-born parents, asthma, obesity, or a life-limiting condition. We also observed an increased risk for MIS-C in children 5-11 years and a reduced risk in adolescents 16-18 years as compared to the reference group of children 0-4 years.

Our overall incidence rate of MIS-C is similar to that in a cohort study by Payne *et al* who calculated incidence rates of MIS-C based on Centers for Disease Control and Prevention COVID-19 and US census data.^7^ They reported the highest risk for MIS-C in children 6-10 years, which is similar to our finding of the highest MIS-C incidence in children 5-11 years. Due to the detailed national health and population registers in Sweden that can be linked to each other on the personal identifier held by each Swedish citizen, we were able to confirm this finding and also expand on exploring sociodemographic factors and assessing the role of comorbidities with regard to MIS-C. We found an association between asthma and MIS-C. Asthma is a disease partly characterized by a dysregulation of the immune system, and children with asthma have been reported to be at increased risk of COVID-19 hospital admissions.^22^ In our study, however, the relative risk increase for MIS-C in children with asthma (aHR <1·5) was relatively modest, and the absolute risk was very low.

Obesity was associated with a doubled risk for MIS-C in the current study. This is in line with previous studies that have reported increased risks both for prolonged COVID-19 and for severe MIS-C in children with obesity.^8^^,^^23^ Of note, we defined obesity according to ICD-10 codes and records of visits to obesity centers, and did not have complete data on height and weight of all study subjects. Finally, children with life-limiting conditions had a tripled increased risk for MIS-C. Life-limiting conditions represent a heterogeneous group of children with a generally increased risk for severe respiratory tract infections and have also been overrepresented in case series of children hospitalized with COVID-19.^24^^,^^25^ Nevertheless, the increased risk for MIS-C has to our knowledge previously not been reported. It is interesting to note that life-limiting conditions are one of the groups currently recommended for COVID-19 immunization from 5 years of age in Sweden.^13^

Children with foreign-born parents had a more than doubled risk for MIS-C. It is possible that this was partly explained by increased disease transmission associated with lower socioeconomic status. Previous studies have indicated that certain ethnicities are overrepresented among MIS-C cases. ^6^^,^^7^^,^^26^ However, we were not able to assess ethnicity as a risk factor for MIS-C in the current study.

We observed a significant shift in age among children with MIS-C throughout the pandemic. The difference was most evident for children 12-15 years of age who were overrepresented during the first wave as compared to the second and third waves. We hypothesize that this was mostly explained by changes in SARS-CoV-2 transmission although the spreading patterns of SARS-CoV-2 in children are not fully known due to limited SARS-CoV-2 testing and changing testing practices throughout the pandemic. The decreased risk for MIS-C seen in adolescents 16-18 years could partly have been explained by the COVID-19 vaccination that started in late summer 2021.

When considering our results for potential public health interventions, it is important to underscore that the absolute risks for MIS-C were extremely low in the current study (overall incidence rate <7 per 100 000 person-years), also in the identified risk populations, and that we were not able to discriminate between an increased risk for MIS-C related to increased SARS-CoV-2 exposure or related to a predisposition for developing MIS-C following infection. Further, the overall prognosis of MIS-C is good provided there is access to aggressive immunosuppressive treatment when needed.^3^ Given that the currently dominating SARS-CoV-2 genotype omicron appears to have lower pathogenicity as compared to previous genotypes, we also might see lower risks for MIS-C in the future.^27^

Further, we did not assess the morbidity attributed directly to primary COVID-19 infection or long COVID in this study.^28^ Antoon *et al* reported increased risks for COVID-19-associated intensive care unit treatment for several comorbidities in a study of children with primary COVID-19.^24^ Nevertheless, children with comorbidities have a higher baseline risk both for being tested for SARS-CoV-2 and for receiving treatment at an intensive care unit. Hence there is a risk that such studies could introduce surveillance bias if not compared with a suitable control group (e.g. children hospitalized for another respiratory virus infection). A British study investigating child deaths associated with SARS-CoV-2 reported that COVID-19 was the likely cause of death in only 40% percent of the deceased children who tested positive for SARS-CoV-2.^1^

The strength of this study was the population-based design, the large study size of > 2 million children with detailed register data on sociodemographic factors and comorbidities including well-defined previously validated measures of comorbidities. There are also some limitations to the study. First, as there has been limited SARS-CoV-2 testing in children, and many children have subclinical SARS-CoV-2 infections, our MIS-C estimates were based on the whole population and not solely the children infected with SARS-CoV-2. It is interesting to note that some of the identified risk factors in this study have previously been associated with an increased risk for severe acute COVID-19. Children born outside Sweden were excluded, including 17 children with MIS-C, due to limited register data on these individuals, which might have introduced selection bias and affects the generalizability of the results.

Second, as MIS-C is a rare outcome, we were not able to accurately investigate less common comorbidities or perform analyses stratified on disease severity to explore potential dose-response effects. When controlling for multiple testing, the associations for asthma and obesity did not reach the significance level. There is also a risk that the lack of significant associations with MIS-C observed for some of the studied risk factors, such as chromosomal anomalies and chronic heart disease was attributed to type 2 error.

Finally, there is a risk that other inflammatory syndromes e.g., Kawasaki disease, toxic shock syndrome or hemophagocytic lymphohistiocytosis were misclassified as MIS-C, as the disease presentation of these conditions partly overlap. Indeed, there have been reports of lower incidence of Kawasaki disease during the COVID-19 pandemic.^29^ Neither is there a universally accepted definition of MIS-C, and there are differences in the classification used in previous studies, not least with regard to the upper age-limit.^6^^,^^7^^,^^30^As we defined MIS-C based on register records, there is also a risk of misclassification bias, yet we believe that the quality register pedSRQ used in the current study has higher accuracy than hospital ICD-10 codes for MIS-C as the MIS-C cases entered in the pedSRQ were continuously discussed by senior pediatric rheumatologists.

To conclude, we report that male sex, age 5-11 years, foreign-born parents, asthma, obesity, and life-limiting condition were associated with increased risk for MIS-C in a large population-based cohort study with adjustments for important confounding factors, but the absolute risks were very low. Knowing these risk populations might facilitate identification of children with MIS-C and potentially guide targeted public health interventions.

## Contributors

SR conceptualized the study, had a leading role in the study design and the register-linkages, performed the statistical analyses, drafted the first version of the manuscript and provided funding for the study. CL participated in the study design, had a leading role in the register-linkages and data management and supervised the statistical analyses. ACH had the leading role in the biweekly meetings discussing new Swedish MIS-C patients and provided MIS-C data to the Swedish Pediatric Rheumatology Quality Register (pedSRQ). AS and ECO, provided valuable input on the study design, had a leading role in the register-linkages, and took part in the data management. AH, TA and BHB provided valuable input on the study design and data analyses. RK and PK provided valuable input on the study design, had leading roles in the biweekly meetings discussing new Swedish MIS-C patients and provided MIS-C data to the pedSRQ. The members of the Swedish Pediatric MIS-C Consortium took part in the biweekly meetings discussing new Swedish MIS-C patients and provided MIS-C data to the pedSRQ. CA conceptualized the study, had a leading role in the study design, and register linkage and provided funding for the study. All authors critically revised, commented on, and approved the final manuscript.

## Data sharing statement

Original data are held by Swedish National Board of Health and Welfare, Statistics Sweden, the Swedish Pediatric Rheumatology Quality Register (pedSRQ) and the Child Obesity Quality Register BORIS. Due to Swedish data storage laws we cannot make the data publicly available. However, any researcher can access the data by obtaining an ethical approval from a regional ethical review board and thereafter asking the registers for the original data. Pseudonymised data may be provided upon requests to the corresponding author, if providing a reasonable proposal and if an appropriate data sharing agreement with Karolinska Institutet can be established.

## Declaration of interests

Samuel Rhedin reports research grants from Ake Wiberg foundation, the Samariten Foundation, the Society of Child Care and Region Stockholm. Catarina Almqvist reports research grants from the Swedish Research Council, the Swedish Heart-Lung Foundation and the Asthma and Allergy Foundation.

## Appendix - List of members of the Swedish pediatric MIS-C consortium

Altman, Maria, Clinical Epidemiology Unit, Department of Medicine Solna, Karolinska Institutet, Sweden. maria.altman@ki.se
Berg, Stefan, Department of Pediatrics, Institute of clinical sciences, Sahlgrenska Academy, University of Gothenburg, Sweden, stefan.berg@pediat.gu.se
Berntson, Lillemor, Department of Women´s and Children´s health, Uppsala University, Sweden, lillemor.berntson@kbh.uu.se
Brodin, Petter, Science for Life Laboratory, Dept. of Women's and Children's Health, Karolinska Institutet, Sweden, petter.brodin@ki.se
Fasth, Anders, Department of Pediatrics, Institute of clinical sciences, Sahlgrenska Academy, University of Gothenburg, Sweden, anders.fasth@pediat.gu.se
Lingman Framme, Jenny, Department of Pediatrics, Institute of clinical sciences, Sahlgrenska Academy, University of Gothenburg, Sweden and Department of Pediatrics, Halland Hospital, Halmstad, Sweden. Jenny.Lingman-Framme@regionhalland.se
Månsson, Bengt, Department of Rheumatology, Clinical Sciences Lund, Lund University, Sweden, bengt.mansson@med.lu.se
Mossberg, Maria, Department of Pediatrics, Clinical Sciences Lund, Lund University, Sweden, maria.mossberg@med.lu.se
Nordenhäll, Charlotta, Sachs’ Children and Youth Hospital, Stockholm, Sweden, charlotta.nordenhall@regionstockholm.se
Palmblad, Karin, Department of Women's and Children's Health, Karolinska Institutet, Sweden, karin.palmblad@regionstockholm.se
Rudolph, André, Pediatric Heart Center Stockholm-Uppsala, Karolinska University Hospital, Stockholm, Sweden and Department of Medicine, Karolinska Institutet, Karolinska University Hospital, Stockholm, Sweden, andre.rudolph@ki.se
Säve-Söderbergh, Eva, Pediatric Clinic, Sunderby sjukhus, Sweden, eva.save-soderbergh@norrbotten.se

## Appendix B Supplementary materials

Image, application 1

Image, application 2
