# Supplementary material for: Risk factors for multisystem inflammatory syndrome in children – A population-based cohort study of over 2 million children
Source: Lancet Reg Health Eur. 2022 Jun 22;19:100443. doi: 10.1016/j.lanepe.2022.100443 (PMC9353212; doi:10.1016/j.lanepe.2022.100443)
Supplement: Supplementary file 1 [file mmc1.docx]

*This translation in Swedish was submitted by the authors and we reproduce it as supplied. It has not been peer reviewed. Our editorial processes have only been applied to the original abstract in English, which should serve as reference for this manuscript*

**Risk-factors for Multisystem Inflammatory Syndrome in Children – a Population-based Cohort Study of over 2 Million Children**

**Translated abstract [Swedish]**

**Bakgrund:** Det är ovanligt att barn blir svår sjuka i akut COVID-19, men infektion med SARS-CoV-2 kan trigga det nya postinfektiösa tillståndet *Multisystem Inflammatory Syndrome in Children* (MIS-C). Ökad kunskap om riskfaktorer för detta tillstånd förbättrar vår förståelse för patogenesen och kan ligga till grund för riktade folkhälsointerventioner. Syftet med studien var att undersöka riskfaktorer för MIS-C med målet att identifiera eventuella riskgrupper hos barn och ungdomar.

**Metod:** I denna registerbaserade kohortstudie inkluderades alla barn och ungdomar <19 år födda i Sverige under perioden 1 mars 2001- 31 december 2020. Data på sociodemografiska faktorer och samsjuklighet (kön, ålder, föräldrars födelseregion och utbildningsnivå, egen astma, autoimmun sjukdom, kromosomala avvikelser, kronisk hjärtsjukdom, kronisk lungsjukdom, fetma och svår kronisk sjukdom, sk ”*life-limiting condition*”) inhämtades från nationella hälsoregister och sociodemografiska register. Som utfall studerades MIS-C diagnos i barnreumaregistret under perioden 1 mars 2020 – 8 december 2021. Hasardkvoter (HR) och 95% konfidensintervall (KI) beräknades med Cox regressionsanalys. Incidensfrekvens per 100 000 person-år beräknades utifrån Poissonfördelning.

**Resultat:** Av 2 117 443 inkluderade barn och ungdomar, utvecklade 253 MIS-C, motsvarande ett incidenstal på 6·8 (95% KI: 6·0-7·6) per 100 000 personår. Manligt kön (HR 1·65, 95% KI: 1·28-2·14), ålder 5-11 år (justerad HR 1·44, 95% KI: 1·06-1·95 med barn 0-4 år som referens), utlandsfödda föräldrar (HR 2·53, 95% KI: 1·93-3·34), astma (justerad HR 1·49, 95% KI: 1·00-2·20), fetma (justerad HR 2·15, 95% KI: 1·09-4·25) och svår kronisk sjukdom (aHR 3·10, 95% CI: 1·80-5·33) identifierades som riskfaktorer för MIS-C. Barn 16-18 år hade en minskad risk för MIS-C (justerad HR 0·45, 95% kI: 0·24-0·85).

**Betydelse:** I denna studie identifierades manligt kön, ålder 5-11 år, utlandsfödda föräldrar, astma, fetma och svår kronisk sjukdom som riskfaktorer för MIS-C. Kunskapen om dessa riskgrupper kan underlätta identifiering av barn med MIS-C och potentiellt vägleda riktade folkhälsointerventioner. Dock var den absoluta risken för MIS-C mycket låg, även bland riskgrupperna.

**Finansiering:** Finansiellt stöd för studien erhölls från Vetenskapsrådet (anslag 2018-02640), Hjärt-Lungfonden (anslag 20210416), Astma- och allergiförbundet, Stiftelsen Åke Wiberg, Stiftelsen Samariten, Sällskapet Barnavård samt från Region Stockholm.
