# Supplementary material for: Risk factors for multisystem inflammatory syndrome in children – A population-based cohort study of over 2 million children
Source: Lancet Reg Health Eur. 2022 Jun 22;19:100443. doi: 10.1016/j.lanepe.2022.100443 (PMC9353212; doi:10.1016/j.lanepe.2022.100443)
Supplement: Supplementary file 2 [file mmc2.docx]

**Online Supplement**

**Risk Factors for Multisystem Inflammatory Syndrome in Children – A Population-based Cohort Study of over 2 Million Children**


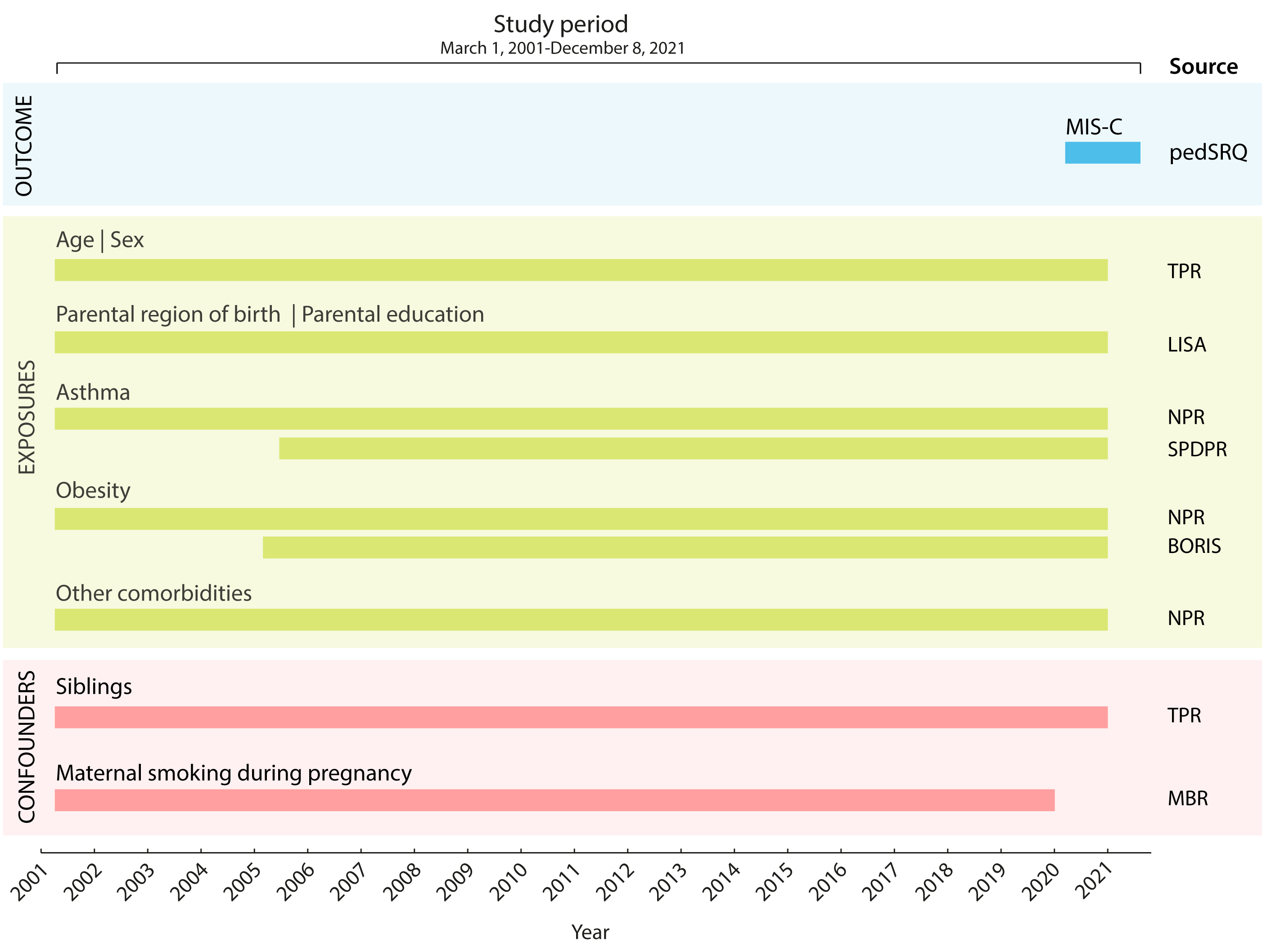


**Supplemental figure 1. Overview of registers.** Abbreviations: BORIS, Child obesity quality register; LISA, the Longitudinal Integratied Database for Labor Market Studies; MBR, Medical Birth Register; NPR, National Patient Register; pedSRQ, Swedish Pediatric Rheumatology Quality Register; SPDR, Swedish Prescribed Drug Register; TPR, Total Population Register.


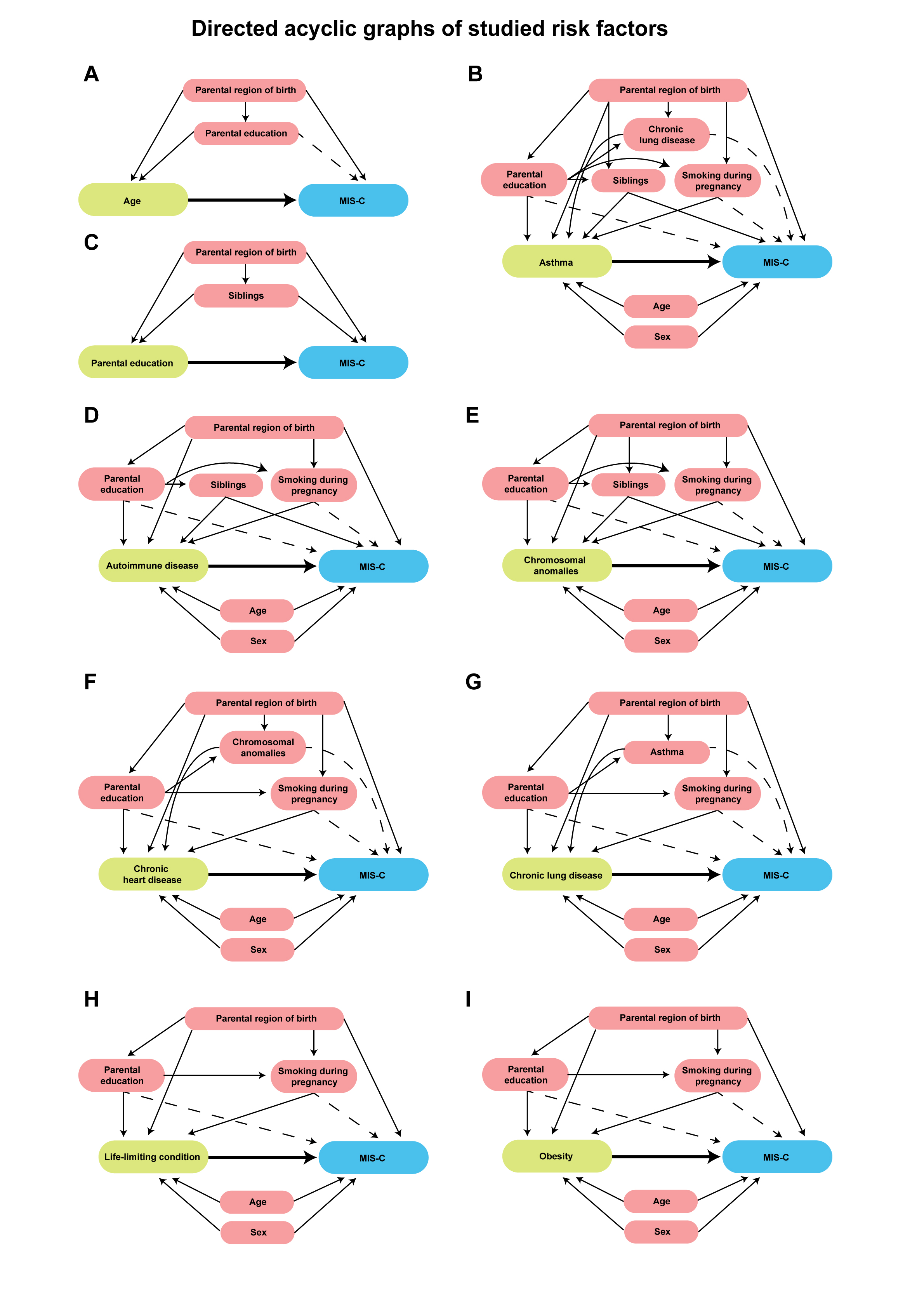


**Supplemental figure 2. Directed acyclic graphs of studied risk factors.** Directed acyclic graphs visualizing relationship between risk factors (green), presumed confounders (pink) and outcome (blue). Dashed arrows indicate presumed relationship with limited data from the literature.

| **Supplemtal table 1 – List of ICD-10 diagnoses used for comorbidity variables** | | |
| --- | --- | --- |
| **Comorbidity variable** | **Disease** | **ICD-10 code** |
| Autoimmune disease | Ankylosing spondylitis | M45 M081 |
|  | Arthritis (Still’s, juvenile, systematic juvenile, idiopathic, seropositive rheumatoid arthritis, other rheumatoid arthritis) | M058 M059 M06 M080 M09 M123 |
|  | Autoimmune encephalitis | G048 |
|  | Autoimmune hepatitis | K73 |
|  | Autoimmune thyroiditis | E063 |
|  | Celiac disease | K900 |
|  | Churg-Strauss syndrome (Eosinophilic granulomatosis with polyangiitis) | M301 |
|  | Crohn’s disease | K50 |
|  | Dermatitis herpetiformis | L122 L130 |
|  | Dermatopolymyositis | M33 |
|  | Glomerulonephritis | N00 N01 N03 N05 |
|  | Vasculitis | M311 M313 M314 M315 M316 M317 |
|  | Guillain-Barre syndrome | G610 |
|  | Interstitial cystitis | N301 |
|  | Kawasaki disease | M303 |
|  | Lupus (systemic lupus erythematosus, discoid lupus erythematosus) | M321 M328 M329 |
|  | Lupus skin | L930 |
|  | Mixed connective tissue disease | M35 |
|  | Multiple sclerosis | G35 |
|  | Myasthenia gravis | G700 |
|  | Myositis | M601 M608 M609 |
|  | Pemphigoid | L12 |
|  | Pemphigus | L10 |
|  | Polyarteritis nodosa (microscopic polyangiitis) | M300 |
|  | Primary adrenocortical insufficiency (other adrenal gland) | E271 |
|  | Primary biliary cirrhosis | K743 |
|  | Psoriasis (vulgaris, psoriatic arthritis, generalized familial pustular psoriasis) | L40 |
|  | Raynaud’s syndrome | I730 |
|  | Rheumatic fever/rheumatic myocarditis | I01 |
|  | Sarcoidosis | D86 |
|  | Schnitzler syndrome (Muckle-Wells syndrome) | L508 |
|  | Scleroderma | M34 |
|  | Sjögren’s syndrome | M350 |
|  | Thyrotoxicosis | E05 |
|  | Type 1 diabetes | E10 |
|  | Ulcerative colitis | K51 |
| Chronic heart disease | Other pulmonary heart diseases | I27 |
|  | Cardiomyopathy | I42 |
|  | Heart failure | I50 |
|  | Cardiovascular disorders originating in the perinatal period | P29 |
|  | Congenital malformations of the circulatory system | Q20-Q28 |
| Chronic lung disease | Cystic fibrosis | E84 |
|  | Bronchiectasis | J47 |
|  | Interstitial lung disorders | J70·4 J84·1 J84·9 |
|  | Chronic respiratory disease originating in the perinatal period | P27 |
|  | Congenital malformations of the lower respiratory tract | Q31-Q33 |
| Chromosomal anomalies | Down syndrome | Q90 |
|  | Other chromosomal anomalies | Q91-Q99 |
| Life-limiting conditions | Tuberculosis of nervous system | A17 |
|  | Creutzfedlt Jakobss disease or syndrome | A81·0 |
|  | Panencephalitits, subacute, sclerosing | A81·1 |
|  | HIV  Malignant Neoplasms | B20-24  C00-C97 |
|  | Benign neoplasm of CNS | D33 |
|  | Neoplasm of brain or CNS | D43 |
|  | Neoplasm of cranio | D44·4 |
|  | Neoplasm of uncertain or unknown behaviour | D48 |
|  | Beta thalassemia | D56·1 |
|  | Constitutional aplastic anaemia | D61·0 |
|  | Aplastic anaemia | D61·9 |
|  | Agranulocytosis | D70 |
|  | Haemophagocytic lymphohistiocytosis  Combined immunodeficiencies | D76·1  D81 |
|  | Di Georges syndrome  Common variable immunodeficiencies | D82·1  D83 |
|  | Cryoglobulinaemia | D89·1 |
|  | Autoimmune polyglandular failure | E31·0 |
|  | Hyperpinealism | E34·8 |
|  | Disorders of tyrosine metabolism | E70·2 |
|  | Disorders of branched-chain amino-acid metabolism and fatty-acid metabolism | E71 |
|  | Other disorders of amino-acid metabolism | E72 |
|  | Other disorders of carbohydrate metabolism | E74 |
|  | Disorders of sphingolipid metabolism and other lipid storage disorders | E75 |
|  | Disorders of glycosaminoglycan metabolism | E76 |
|  | Disorders of glycoprotein metabolism | E77 |
|  | Lesch-Nyhan syndrome | E79·1 |
|  | Disorders of copper metabolism | E83·0 |
|  | Cystic fibrosis | E84 |
|  | Disorders of plasma-protein metabolism | E88·0 |
|  | Lipodystrophy | E88·1 |
|  | Acquired aphasia with epilepsy | F80·3 |
|  | Rett syndrome | F84·2 |
|  | Huntington disease | G10 |
|  | Early-onset cerebellar ataxia | G11·1 |
|  | Cerebellar ataxia with defective DNA repair | G11·3 |
|  | Spinal muscular atrophy and related syndromes | G12 |
|  | Parkinson disease | G20 |
|  | Hallervorden-Spatz disease | G23·0 |
|  | Other specified degenerative diseases of basal ganglia  Other specified degenerativve diseases of nervous system | G23·8  G31·8 |
|  |  |  |
|  | Degenerative disease of nervous system, unspecified | G31·9 |
|  | Multiple sclerosis | G35 |
|  | Other generalized epilepsy and epileptic syndromes  Special epileptic syndromes | G40·4  G40·5 |
|  |  |  |
|  | Hereditary motor and sensory neuropathy | G60·0 |
|  | Refsum disease | G60·1 |
|  | Congenital and developmental myasthenia  Myoneural disorder, unspecified | G70·2  G70·9 |
|  |  |  |
|  | Muscular dystrophy | G71·0 |
|  | Myotonic disorders | G71·1 |
|  | Congenital myopathies | G71·2 |
|  | Mitochondrial myopathy | G71·3 |
|  | Spastic quadriplegic cerebral palsy | G80·0 |
|  | Other cerebral palsy | G80·8 |
|  | Flaccid tetraplegia | G82·3 |
|  | Spastic tetraplegia | G82·4 |
|  | Tetraplegia, unspecified | G82·5 |
|  | Encephalopathy, unspecified | G93·4 |
|  | Cerebral oedema | G93·6 |
|  | Reye syndrome | G93·7 |
|  | Conjunctival degenerations and deposits | H11·1 |
|  | Hereditary retinal dystrophy | H35·5 |
|  | Other paralytic strabismus | H49·8 |
|  | Acute myocardial infarction | I21 |
|  | Intracerebral haemorrhage in brain stem | I61·3 |
|  | Portal vein thrombosis | I81 |
|  | Other disorders of lung | J98·4 |
|  | Acute vascular disorders of intestine | K55·0 |
|  | Vascular disorder of intestine, unspecified | K55·9 |
|  | Hepatic veno-occlusive disease | K76·5 |
|  | Other specified diseases of pancreas | K86·8 |
|  | Wegener granulomatosis | M31·3 |
|  | Systemic lupus erythematosus | M32·1 |
|  | Osteolysis  Acute renal failure  Chronic kidney disease  Unspecified kidney failure  Other disorders resulting from impaired renal tubular function | M89·5  N17  N18  N19  N25·8 |
|  | Cerebral haemorrhage due to birth injury | P10·1 |
|  | Unspecified brain damage due to birth injury | P11·2 |
|  | Severe birth asphyxia | P21·0 |
|  | Respiratory failure of newborn | P28·5 |
|  | Congenital rubella syndrome | P35·0 |
|  | Congenital cytomegalovirus infection | P35·1 |
|  | Other congenital viral diseases | P35·8 |
|  | Congenital toxoplasmosis | P37·1 |
|  | Intracerebral (nontraumatic) haemorrhage of fetus and newborn | P52·4 |
|  | Subarachnoid (nontraumatic) haemorrhage of fetus and newborn | P52·5 |
|  | Intracranial (nontraumatic) haemorrhage of fetus and newborn, | P52·9 |
|  | Hydrops fetalis not due to haemolytic disease | P83·2 |
|  | Neonatal cerebral leukomalacia | P91·2 |
|  | Hypoxic ischaemic encephalopathy of newborn | P91·6 |
|  | Congenital renal failure | P96·0 |
|  | Anencephaly | Q00·0 |
|  | Encephalocele | Q01 |
|  | Atresia of foramina of Magendie and Luschka | Q03·1 |
|  | Congenital hydrocephalus | Q03·9 |
|  | Congenital malformations of corpus callosum | Q04·0 |
|  | Holoprosencephaly | Q04·2 |
|  | Other reduction deformities of brain | Q04·3 |
|  | Septo-optic dysplasia | Q04·4 |
|  | Congenital cerebral cysts | Q04·6 |
|  | Congenital malformation of brain, unspecified | Q04·9 |
|  | Arnold-Chiari syndrome | Q07·0 |
|  | congenital malformations of trachea | Q32·1 |
|  | Hypoplasia and dysplasia of lung | Q33·6 |
|  | Diverticulum of oesophagus | Q39·6 |
|  | Congenital absence, atresia and stenosis of duodenum | Q41·0 |
|  | Congenital absence, atresia and stenosis of small intestine, part unspecified | Q41·9 |
|  | Persistent cloaca | Q43·7 |
|  | Atresia of bile ducts  Other congential malformations of bile ducts | Q44·2  Q44·5 |
|  | Other congenital malformations of liver | Q44·7 |
|  | Renal agenesis, bilateral | Q60·1 |
|  | Potter syndrome | Q60·6 |
|  | Renal dysplasia | Q61·4 |
|  | Cystic kidney disease | Q61·9 |
|  | Congenital posterior urethral valves | Q64·2 |
|  | Arthrogryposis multiplex congenita | Q74·3 |
|  | Other specified congenital malformations of limb(s) | Q74·8 |
|  | Craniosynostosis | Q75·0 |
|  | Short rib syndrome | Q77·2 |
|  | Chondrodysplasia punctata | Q77·3 |
|  | Achondroplasia | Q77·4 |
|  | Osteogenesis imperfecta | Q78·0 |
|  | Metaphyseal dysplasia | Q78·5 |
|  | Exomphalos | Q79·2 |
|  | Gastroschisis | Q79·3 |
|  | Harlequin fetus | Q80·4 |
|  | Epidermolysis bullosa | Q81 |
|  | Xeroderma pigmentosum | Q82·1 |
|  | Ectodermal dysplasia | Q82·4 |
|  | Other phakomatoses, not elsewhere classified | Q85·8 |
|  | Fetal alcohol syndrome | Q86·0 |
|  | Congenital malformation syndromes predominantly affecting facial appearance | Q87·0 |
|  | Congenital malformation syndromes predominantly associated with short stature | Q87·1 |
|  | Congenital malformation syndromes predominantly involving limbs | Q87·2 |
|  | Other specified congenital malformation syndromes, not elsewhere classified | Q87·8 |
|  | Bone-marrow transplant rejection | T86·0 |
|  | Heart transplant failure and rejection | T86·2 |
|  | Palliative care | Z51·5 |
| Obesity | Overweight, obesity and other hyperalimentation | E60-E65 |
|  | Record of obesity class 1-III in BORIS |  |

| **Supplemental table 2 – Hazard ratios for different age groups and MIS-C according to COVID-19 wave** | |
| --- | --- |
| **Age groups** | **MIS-C** |
|  | HR (95% CI) |
| First COVID-19 wave |  |
| 0-4 years | Ref |
| 5-11 years | 1·10 (0·54-2·24) |
| 12-15 years | 2·20 (1·07-4·53) |
| 16-18 years | 0·67 (0·22-2·01) |
| Second and third COVID-19 wave |  |
| 0-4 years | Ref |
| 5-11 years | 1·43 (1·02-2·01) |
| 12-15 years | 0·92 (0·59-1·44) |
| 16-18 years | 0·33 (0·16-0·71) |

Abbreviations: CI, confidence interval; HR, hazard ratio.

| **Supplemental table 3 – Hazard ratios and p-values for MIS-C for each studied risk factor** | | | | |
| --- | --- | --- | --- | --- |
|  | **MIS-C** | | | |
| **Risk-factor** | HR (95% CI) | p-value | adjusted HR (95% CI) | p-value |
| Male sex | 1·65 (1·28-2·14) | <0·001 | NA | NA |
| AGE |  | <0·001 |  | <0·001 |
| 0-4 years | Ref |  | Ref |  |
| 5-11 years | 1·37 (1·01-1·85) | 0·046 | 1·44 (1·06-1·95) | 0·046 |
| 12-15 years | 1·18 (0·81-1·71) | 0·390 | 1·31 (0·90-1·90) | 0·163 |
| 16-18 years | 0·40 (0·22-0·75) | 0·004 | 0·45 (0·24-0·85) | 0·013 |
| PARENTAL EDUCATION |  |  |  |  |
| Primary school | Ref |  | Ref |  |
| Secondary school | 0·87 (0·49-1·55) | 0·636 | 1·19 (0·66-2·16) | 0·558 |
| Tertiary education | 0·76 (0·43-1·34) | 0·340 | 1·12 (0·62-2·02) | 0·701 |
| PARENTAL REGION OF BIRTH |  |  |  |  |
| Both parents born in Sweden | Ref |  | NA | NA |
| One parent born in Sweden | 1·41 (0·98-2·03) | 0·063 | NA | NA |
| Both parents born abroad | 2·54 (1·93-3·34) | <0·001 | NA | NA |
| COMORBIDITIES |  |  |  |  |
| Asthma | 1.44 (1.00-2.08) | 0·051 | 1.49 (1.00-2.20) | 0·048 |
| Autoimmune disease | 1·22 (0·45-3·28) | 0·693 | 1·28 (0·41-4·01) | 0·676 |
| Chromosomal anomalies | 3·07 (0·76-12·33) | 0·115 | 3·43 (0·85-13·82) | 0·083 |
| Chronic heart disease | 1·50 (0·77-2·92) | 0·230 | 1·59 (0·79-3·18) | 0·187 |
| Chronic lung disease | 0·74 (0·10-5·26) | 0·761 | 0·73 (0·10-5·24) | 0·758 |
| Life-limiting condition | 2·72 (1·59-4·66) | 0·046 | 3·10 (1·80-5·33) | <0·001 |
| Obesity | 2·22 (1·18-4·21) | 0·014 | 2·15 (1·09-4·25) | 0·027 |

Bonferroni corrected p-value threshold=0.0045 assuming 11 tests and an overall significance level of 5%. Abbreviations: HR, hazard ratio; CI, confidence interval; MIS-C, multisystem inflammatory syndrome; NA, not applicable.

| **Supplemental table 4 – Hazard ratios for MIS-C after exclusion of children with life-limiting condition** | | |
| --- | --- | --- |
|  | **MIS-C** | |
| **Risk-factor** | HR (95% CI) | adjusted HR (95% CI) |
| Male sex | 1·60 (1·23-2·08) | NA |
| AGE |  |  |
| 0-4 years | Ref | Ref |
| 5-11 years | 1·40 (1·02-1·90) | 1·47 (1·08-2·01) |
| 12-15 years | 1·04 (0·70-1·55) | 1·16 (0·78-1·73) |
| 16-18 years | 0·39 (0·20-0·74) | 0·44 (0·23-0·84) |
| PARENTAL EDUCATION |  |  |
| Primary school | Ref | Ref |
| Secondary school | 0·88 (0·48-1·61) | 1·23 (0·66-2·29) |
| Tertiary education | 0·78 (0·43-1·41) | 1·19 (0·65-2·18) |
| PARENTAL REGION OF BIRTH |  |  |
| Both parents born in Sweden | Ref | NA |
| One parent born in Sweden | 1·53 (1·06-2·20) | NA |
| Both parents born abroad | 2·62 (1·97-3·47) | NA |
| COMORBIDITIES |  |  |
| Asthma | 1.47 (1.00-2.14) | 1·53 (1·02-2·30) |
| Autoimmune disease | 1·35 (0·50-3·62) | 1·47 (0·47-4·63) |
| Chromosomal anomalies | - | - |
| Chronic heart disease | 0·68 (0·22-2·13) | 0·81 (0·26-2·55) |
| Chronic lung disease | 1·02 (0·14-7·24) | 1·00 (0·14-7·14) |
| Obesity | 2·16 (1·10-4·22) | 2·12 (1·03-4·36) |

Abbreviations: HR, hazard ratio; CI, confidence interval; MIS-C, multisystem inflammatory syndrome; NA, not applicable.
